# Preoperative T staging using CT colonography with multiplanar reconstruction for very low rectal cancer

**DOI:** 10.1186/s12885-017-3756-9

**Published:** 2017-11-14

**Authors:** Dai Shida, Gen Iinuma, Akira Komono, Hiroki Ochiai, Shunsuke Tsukamoto, Mototaka Miyake, Yukihide Kanemitsu

**Affiliations:** 1 Colorectal Surgery Division, National Cancer Center Hospital, 5-1-1 Tsukiji, Chuo-ku, Tokyo, 1040045 Japan; 2 Department of Diagnostic Radiology, National Cancer Center Hospital, 5-1-1 Tsukiji, Chuo-ku, Tokyo, 1040045 Japan

**Keywords:** CT colonography, Multiplanar reconstruction (MPR), Lower rectal cancer, Preoperative T staging

## Abstract

**Background:**

Preoperative T staging of lower rectal cancer is an important criterion for selecting intersphincteric resection (ISR) or abdominoperineal resection (APR) as well as selecting neoadjuvant therapy. The aim of this study was to evaluate the accuracy of preoperative T staging using CT colonography (CTC) with multiplanar reconstruction (MPR), in which with the newest workstation the images can be analyzed with a slice thickness of 0.5 mm.

**Methods:**

Between 2011 and 2013, 45 consecutive patients with very low rectal adenocarcinoma underwent CTC with MPR. The accuracy of preoperative T staging using CTC with MPR was evaluated. The accuracy of preoperative T staging using MRI in the same patient population (34 of 45 patients) was also examined.

**Results:**

Overall accuracy of T staging was 89% (41/45) for CTC with MPR and 71% (24/34) for MRI. CTC with MPR was particularly sensitive for pT2 tumors (82%; 14/17), whereas MRI tended to overstage pT2 tumors and its sensitivity for pT2 was 53% (8/15).

**Conclusions:**

CTC with MPR, with an arbitrary selection, could be aligned to the tumor axis and better demonstrated tumor margins consecutively including the deepest section of the tumor. The accuracy of T2 and T3 staging using CTC with MPR seemed to surpass that of MRI, suggesting a potential role of CTC with MPR in preoperative T staging for very low rectal cancer.

## Background

Preoperative T staging of lower rectal cancer is an important criterion for selecting intersphincteric resection (ISR) or abdominoperineal resection (APR) as well as selecting neoadjuvant therapy. In addition to the infiltration of the external sphincter and the levator muscles, preoperative T staging, which has close relation with circumferential resection margin (CRM), is one of criterion for judgment to select ISR or APR for patients with very low rectal cancer. Imaging modalities currently used in the staging of rectal cancer include magnetic resonance imaging (MRI) [1], endoscopic ultrasonography (EUS) [2, 3], and computed tomography (CT).

Preoperative T staging using multi-detector row CT (MDCT) for gastric cancers, categorized according to the AJCC TNM classification, has been well-established [4–7]. For rectal cancer, preoperative local staging using MDCT with multiplanar reconstruction (MPR) has recently been reported [8, 9]. Ahmetoglu et al. [9] reported an 86% overall accuracy of T staging using MDCT with MPR in 37 patients with rectal cancer. Kobayashi et al. [8] reported that the overall accuracy of T staging using MDCT with MPR was 61% (44/72) in 72 patients with rectal cancer, with the accuracy for pT2 tumors being the lowest (4/18) [8], suggesting that the biggest challenge lies in distinguishing T2 from T3 preoperatively. Differentiation between T2 and T3 tumors is very important, because this leads to different preoperative staging; T2 N0 is considered stage I, whereas T3 N0 is considered stage II. Thus, preoperative T staging needs to be improved in order to allow for better differentiation, especially between T2 and T3 tumors.

Recently, marked developments in CT colonography (CTC) with MPR and three-dimensional (3D) images, which are reconstructed using the newest image workstation, have made possible the acquisition of more accurate thin slice images and optional images. This allows for the delineation of the spatial relationship between the tumor and its extramural layers. All CTC images with MPR are reconstructed with a 1.0 mm effective thickness at 0.8 mm intervals, and the slices are transferred to the workstation where the virtual line and virtual images can be created. With the newest post-processing workstation, MPR images can be analyzed with a slice thickness of 0.5 mm, whereas MRI can only analyze slice thicknesses up to 2 mm. In addition to performing MPR from thin slice images, the current state-of-the-art workstations has the ability to merge 2D MPR and CPR (curved planar reconstruction) views with Virtual Endoscopy images (allowing to precisely 3D locate even small lesions for optimal correlation with pathological specimens), and their seamless integration with imaging modalities via modern PACS (picture archiving and communication system) infrastructure allows to simplify and speed up diagnostic workflow. Accordingly, thinner slices obtained by CTC with MPR using the advanced workstation are expected to improve the prediction of T2 and T3.

The aim of this study was to evaluate the accuracy, sensitivity, specificity, positive predictive value (PPV), and negative predictive value (NPV) of preoperative T staging using CTC with MPR.

## Methods

### Study population

Between 2011 and 2013, 45 consecutive patients with very low rectal adenocarcinoma located within 5 cm from the anal verge, who did not receive neoadjuvant chemotherapy or radiotherapy, underwent CTC with MPR at the National Cancer Center Hospital, Tokyo. We evaluated the accuracy of preoperative T staging using CTC with MPR in those 45 patients with very low rectal cancer. The accuracy of preoperative T staging using MRI was also evaluated in 34 of the 45 patients, excluding 11 who did not undergo MRI examination. Patients provided written informed consent and underwent CTC on the same day as conventional colonoscopy. Biopsies subsequently confirmed the presence of colorectal adenocarcinoma. All patients underwent surgical resection within one month of CTC examination. This study was approved by the National Cancer Center Hospital institutional review boards (IRB) (IRB code: 2015–032).

### CTC procedure

CTC procedures have been described previously [10, 11]. Patients underwent MDCT using an intravenous contrast medium immediately after total colonoscopy without stool tagging. Thus, each patient received a bowel preparation in the form of polyethylene glycol solution (Fusimi Pharma; Kagawa, Japan) before colonoscopy. Before the CTC procedure, an antiperistaltic agent (20 mg scopolamine butylbromide) was intramuscularly administered and an enema tube inserted into the rectum in the left lateral decubitus position. Patients received automated carbon dioxide (PROTOCO2L Insufflator). The enema tube remained in the rectum during examination. Initially, CTC was performed with patients in the prone position. The procedure was subsequently repeated with patients in the supine position.

CTC was performed with a 64× multidetector CT scanner (Aquilion, Toshiba Medical Systems, Tokyo, Japan). Scans were obtained through the abdomen and pelvis using the following parameters: 120 kV; 200–400 mA with automatic exposure control; 64 rows × 0.5 mm collimation; and helical pitch, 53 (pitch factor 0.828). Each patient received an intravenous bolus injection of 150 mL (or 120 mL for the patients weighing 40–50 kg, or 100 ml for the patients weighing 40 kg or less) of a contrast medium (iohexol 350; Omnipaque, Daiichi-Sankyo Pharmaceutical, Tokyo, Japan), from a power injector at a rate of 3 mL/s through a 20-gauge plastic IV catheter placed in an antecubital vein. The entire abdomen was scanned with a scan delay of 50 s (a mixed late arterial/early enteric phase for detecting marginal arteries) after introduction of the contrast material. All images were reconstructed with a 0.5 mm effective thickness at 0.5 mm intervals, and slices were transferred to an image workstation (Ziostation2, Ziosoft Inc., Tokyo, Japan) to generate 3D images.

### Interpretation of CTC

Since the lower rectum lacks the serosa, for lower rectal cancer, T2 and T3 are differentiated simply based on the extramural layer being smooth or not; that is, tumors with a smooth extramural layer are considered T2, and those with a rough extramural layer are considered T3. Representative cases for T2 and T3 stages are provided below.

### Case 1

**Fig. 1:**
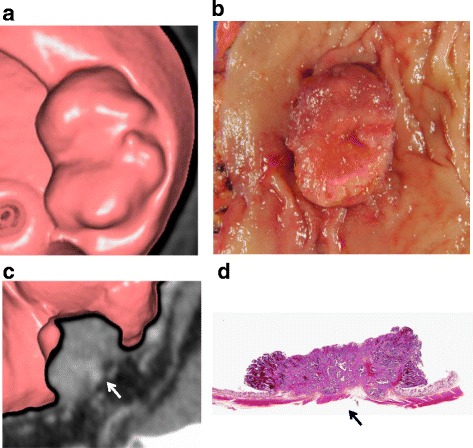
Patient in the sixties with T2 lower rectal cancer. **a** CTC imaging of the tumor with MPR revealing a tumor 30 mm in size. **b** The macroscopic appearance of the tumor showing an irregular ulceration and clear marginal swelling. **c** MPR images showing the tumor not reach the marginal vessels (arrow) with smooth outer border. **d** A resected specimen showing tumor invasion to the muscularis propria, but without marginal vessel involvement (arrow), resulting in pathological stage T2

Figure 1 shows a patient in the sixties with T2 lower rectal cancer. CTC imaging with MPR revealed a tumor 30 mm in size (A), which was highly consistent with the macroscopic appearance of the tumor with an irregular ulceration and clear marginal swelling (B). The tumor did not reach the marginal vessels (arrow) and was contained in the extramural layer, according to MPR images (C). Thus, preoperative T staging by CTC was T2. A resected specimen at the same level as captured by the MRP image revealed tumor invasion to the muscularis propria but without marginal vessel involvement (arrow). Pathologically, the tumor was staged as T2 (D).

### Case 2

**Fig. 2:**
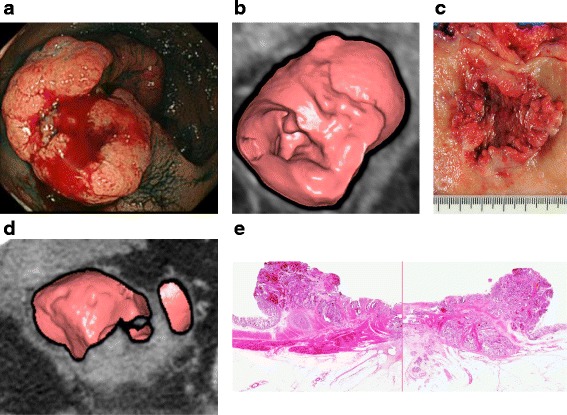
Patient in the fifties with T3 lower rectal cancer. **a** Endoscopic appearance showing irregular ulceration and clear marginal swelling. **b** CTC imaging with MPR revealing a tumor 50 mm in size. **c** Macroscopic appearance showing irregular ulceration and clear marginal swelling. **d** MPR images showing the tumor with an irregular extramural layer. Thus, preoperative T staging by CTC was T3. **e** revealed tumor invasion beyond the muscularis propria, and the lesion was pathologically staged as T3

Figure 2 shows a patient in the fifties with T3 lower rectal cancer. CTC imaging of the tumor with MPR (B) revealed a tumor 50 mm in size, which was highly consistent with the endoscopic (A) and macroscopic (C) appearance showing irregular ulceration and clear marginal swelling. The MPR images revealed a tumor with an irregular extramural layer (D). Thus, preoperative T staging by CTC was T3. A resected specimen at the same level captured by the MRP image revealed tumor invasion beyond the muscularis propria, and the lesion was pathologically staged as T3 (E).

### Image analysis of CTC

Following above, blind interpretation of CTC by two radiologists, with 20 years and 10 years of experience in abdominal CT interpretation, were obtained preoperatively. Contrast-enhanced transverse CT images were initially assessed, followed by a second review combining both contrast enhanced transverse images and MPR. T staging was performed for CTC using MPR images generated from a mixed late arterial/early enteric phase CT images. The interpretation was also confirmed by at least four experienced surgeons at a preoperative conference in the colorectal surgery division of our hospital. Differences in assessment were resolved by consensus. Depth of transmural invasion was categorized as ctT1, ctT2, ctT3, and ctT4, according to the AJCC TNM classification.

### MRI procedure

MRI procedures of our hospital for rectal cancer have been described previously [1, 12]. The patients received a 150-ml glycerin enema before examination and were placed in a supine, head-first position. No air insufflation was used, but an intramuscular antispasmodic was administered. We used a 3-T whole-body system (MAGNETOM Trio, A Tim System 3 T, Siemens Healthineers Japan, Tokyo, Japan). Initially, sagittal T2-weighted fast spin-echo images (repetition time/echo time, 4500 ms/86 ms; echo-train length, 11; slice thickness, 3 mm; gap, 0.6 mm; signal averages, 1; matrix, 384 × 384; field of view, 23 cm × 23 cm) of the pelvis were obtained. These images were used to plan T2-weighted thin-section axial imaging. Axial T2-weighted thin-section fast spin-echo images (repetition time/echo time, 4000 ms/74 ms; echo-train length, 9; slice thickness, 3 mm; gap, 0 mm; signal averages, 1; matrix, 384 × 384; field of view, 23 cm × 23 cm) of the pelvis were then obtained.

### Interpretation of MRI

Two experienced radiologists with 20 years and 10 years of experience in abdominal MRI interpretation and who was completely blinded to lesion size, macroscopic features, and stage of colorectal cancers, interpreted each high resolution MRI image on the workstation monitor, as described previously [1, 12]. Depth of transmural invasion was categorized as mrT1, mrT2, mrT3, and mrT4, according to the AJCC TNM classification and was assessed based on reported criteria [1]. The presence of spiculation within fat alone was not regarded as sufficient evidence of extramural invasion. Small interruptions of the outer contours of the muscle coat were also not considered sufficient to diagnose a T3 lesion. Five patients who received endoscopic resection before MRI and six patients who did not undergo an examination by MRI were excluded from the evaluation of accuracy for transmural tumor invasion depth.

### Statistical analysis

All statistical analyses were performed using the JMP12 software program (SAS institute Japan LTD., Tokyo, Japan).

## Results

The accuracy, sensitivity, specificity, PPV, and NPV of preoperative T staging using CTC with MPR in 45 patients were examined. Patient characteristics are shown in Table 1. These of preoperative T staging using MRI in the same patient population (34 of 45 patients) was also examined.

**Table 1:** Patient characteristics

Gender	Male	27
	Female	18
Age (years)		65 (36–81)
BMI (kg/m^2^)		21.8 (14.2–36.2)
Tumor size (cm)		4.0 (1.0–9.5)
Tumor location from anal verge (cm)		3.5 (1.0–5.0)
Pathological T	T1	6
	T2	17
	T3	21
	T4	1
Stage	I	15
	II	8
	III	21
	IV	1

As shown in Table 2, overall sensitivity of T staging using MRI was 71% (24/34). The sensitivity of MRI was very high for pT3 tumors (94%; 15/16) as well as pT4 tumors (100%; 1/1). In contrast, the sensitivity of MRI was low for pT2 tumors (53%; 8/15). Staging errors were mainly due to overstaging of T2 tumors.

**Table 2:** Comparison of sensitivity as well as accuracy, specificity, PPV,NPV of preoperative T staging for lower rectal cancer using CTC with MPR and MRI

CTC with MPR (N=45)								MRI (*n*=34)					
	ctT1	ctT2	ctT3	ctT4	n	sensitivity		mrT1	mrT2	mrT3	mrT4	n	sensitivity
pT1	5		1		6	83%	pT1	0	1	1		2	0%
pT2	2	14	1		17	82%	pT2		8	7		15	53%
pT3			21		21	100%	pT3		1	15		16	94%
pT4				1	1	100%	pT4				1	1	100%
	7	14	23	1	45	91%		0	10	23	1	34	71%
			pT1				pT2			pT3			
			CTC (*n*=6)		MRI (*n*=2)		CTC (*n*=17)		MRI (*n*=15)	CTC (*n*=21)		MRI (*n*=16)	
Accuracy			93% (42/45)		94% (32/34)		93% (42/45)		74% (25/34)	96% (43/45)		74% (25/34)	
Sensitivity			83% (5/6)		0% (0/2)		82% (14/17)		53% (8/15)	100% (21/21)		94% (15/16)	
Specificity			95% (37/39)		100% (32/32)		100% (28/28)		89% (17/19)	92% (22/24)		56% (10/18)	
PPV			71% (5/7)		- (0/0)		100% (14/14)		80% (8/10)	91% (21/23)		65% (15/23)	
NPV			97% (37/38)		94% (32/34)		90% (28/31)		71% (17/24)	100% (22/22)		91% (10/11)	

Preoperative T staging using CTC with MPR revealed an overall sensitivity of 91% (41/45) (Table 2). In particular, the sensitivity for pT2 tumors was high at 82% (14/17), compared to that of MRI (53%) (*p* = 0.37).

Table 2 also shows the accuracy, sensitivity, specificity, PPV, and NPV of preoperative T staging for CTC and MRI by stage. One case of T4b, which had accurate T staging based on both CTC and colonoscopy, was excluded from the Table.

**Fig. 3:**
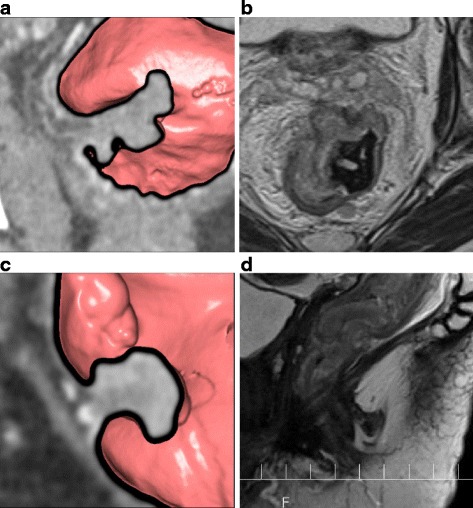
Two representative cases of pathological T2 tumors which were staged correctly by CTC with MPR but overstaged by MRI. (A, B): Patient in the seventies with pathological T2 lower rectal cancer, 45 mm in size. **a** Consecutive CTC imaging with MPR, aligned to the tumor axis, revealed a tumor with a smooth extramural layer, which was considered T2 tumor. **b** Axial T2-weighted MR imaging showed a tumor invading beyond the muscularis propria at right ventral side, which was considered T3 tumor. (C, D): Patient in the seventies with pathological T2 lower rectal cancer, 30 mm in size. **c** Consecutive CTC imaging with MPR, aligned to the tumor axis, revealed a tumor with a smooth extramural layer, which was considered T2 tumor. **d** Sagittal T2-weighted MR imaging showed a tumor invading beyond the muscularis propria at left dorsal side, which was considered T3 tumor

In pT2 cases, similar to the sensitivity mentioned above, the accuracy was 93% for CTC, which was significantly higher compared to 74% for MRI (*p* = 0.02) (Table 2). In addition, the specificity, PPV, and NPV were all higher for CTC than MRI. Among fifteen pT2 patients, preoperative MRI staging was mrT2 in 8 patients and mrT3 in 7 patients. Thus, MRI tended to overstage pT2 tumors and its sensitivity for pT2 was 53% (8/15), which is same as our previous report [1]. Figure 3 showed two representative cases of pT2 tumors which were staged correctly by CTC with MPR but overstaged by MRI.

In pT3 cases, the accuracy of CTC was also higher compared to MRI (96% and 74%, respectively), as was the specificity (92% and 56%, respectively) (Table 2). In addition, the sensitivity, PPV, and NPV were all higher for CTC compared to MRI.

Taken together, in the preoperative diagnosis of lower rectal cancer, T staging using CTC appears to be superior to MRI, particularly in distinguishing between T2 and T3 tumors.

## Discussion

Recent advances in CTC with MPR using an image workstation have enabled acquisition of very thin slice images with high resolution, making possible the accurate visualization of the extramural layer. In the present study, all images of CTC with MPR were reconstructed with a 1.0 mm effective thickness at 0.8 mm intervals, and the slices were transferred to a workstation where the virtual line and images were created. With this advanced workstation, we were able to analyze MPR images with a slice thickness of 0.5 mm, whereas MRI only allows analysis with a slice thickness of 2 mm. Thus, with further developments in CTC with MPR, it might be possible to overcome the difficulty of preoperative diagnosis pertaining to T2 and T3 staging. Indeed, in our cases, the overall sensitivity of T staging using CTC with MPR was 91%. Notably, the sensitivity for pT2 tumors was 82%, demonstrating a marked improvement compared to MRI (53%). Thus, in the preoperative diagnosis of lower rectal cancer, T staging using CTC appears to be superior to MRI, particularly in distinguishing between T2 and T3 tumors. Whereas this study was limited by small subgroups from a single institution, CTC with MPR using an advanced workstation appears to be a promising modality for preoperative T staging.

The imaging modalities that are currently used in the staging of rectal cancer include MRI and CT. MRI is a reliable technique in the staging of rectal cancer because of its inherently high soft tissue contrast resolution. Akasu et al. reported an overall sensitivity rate for transmural invasion depth of 84% [1]. In addition, the sensitivity for pT3 was excellent (96%); however, interpretation of these results requires caution, since the sensitivity rate dropped to 56% (14/25) for pT2 tumors (*n* = 25) [1]. Thus, even MRI does not always provide an accurate means to distinguish between T2 and T3. In a meta-analysis of previous reviews, the sensitivity and specificity of MRI for perirectal tissue invasion were 82% and 76%, respectively [13]. In our study the sensitivity and specificity of CTC with MPR using the advanced image workstation were higher than those of MRI reported previously. Compared with MRI, CT is widely available, less expensive, and less time-consuming, and is commonly used to detect distant metastasis. The introduction of MDCT has allowed faster scanning, thinner slices, and markedly improved imaging resolution, especially for MPR images. These advantages may increase the accuracy of MDCT with MPR and 3D images for T staging of gastrointestinal tumors. Thus, CTC with MPR is a valuable tool for preoperative T staging of lower rectal cancer, with an inherently high accuracy to detect distant metastasis [14].

MR has better contrast resolution, thus T4b cases might be better detected on MRI. In our previous report [1], pathological T4b cases were 14 cases and these were all correctly staged as mriT4b by MRI. In the present study, although the number of patients with T4b disease is small, we correctly staged one patient with T4b diseases and no false positive and false negative results by MRI.

## Conclusions

In conclusion, this study showed CTC with MPR, with an arbitrary selection, could be aligned to the tumor axis and better demonstrated tumor margins consecutively including the deepest section of the tumor, thus MPR of CTC as a post-processing tool added diagnostic value over MRI. Especially, the accuracy of CTC with MPR for the staging of T2 and T3 tumors was considerably high, suggesting a potential role of CTC with MPR in preoperative T staging for very low rectal cancer.

## Abbreviations

3D: Three-dimensional
APR: Abdominoperineal resection
CRM: Circumferential resection margin
CTC: CT colonography
ISR: Intersphincteric resection
MDCT: Multi-detector row CT
MPR: Multiplanar reconstruction
NPV: Negative predictive value
PPV: Positive predictive value
